# Improvement of solubility and pharmacokinetic profile of hepatoprotector icariin through complexation with HP-*γ*-cyclodextrin

**DOI:** 10.3389/fphar.2023.1138686

**Published:** 2023-03-22

**Authors:** Yili Ding, Bo Yu, Shuzhen Zhou, Charles Ding, Zhiyuan Zhang, Shufeng Xu, Zhe Xu

**Affiliations:** ^1^ College of Science and Technology, Wenzhou-Kean University, Wenzhou, Zhejiang, China; ^2^ Life Science Department, Foshan University, Foshan, Guangdong, China; ^3^ Eastern Along Pharmaceutical Co., Ltd., Foshan, Guangdong, China; ^4^ Keck School of Medicine of USC, Los Angeles, CA, United States

**Keywords:** icariin, inclusion complex, water solubility, *in vitro* and *in vivo* PK study, cyclodextrin

## Abstract

Icariin as a hepatoprotector from *Herba epimedii* can expand the cardiovascular and cerebral blood vessels, promote hematopoietic functions, enhance the immune system and show anti-liver tumor activities. However, its low solubility (0.02 mg/mL) limits its clinical applications as food and medical supplements. Through complexation with HP-*γ*-cyclodextrin by using a trace amount of water-soluble polymer, the water solubility of icariin was increased by 654 times, which is the best result to date for the water solubility study of icariin. In an *in vitro* pharmacokinetic study, the complexation increased the dissolution rate of icariin by 80 times, and the icariin complex can be 100% released in the first few minutes. Through complexation, in an *in vivo* dog pharmacokinetic study, the *C*_*max*_ of icariin was increased about 5 times, the AUC_0-120_ was increased about 20 times and the clearance of icariin was changed from 11.17 L/h/kg to 0.65 L/h/kg. The half-life time was changed from 0.68 h to 6.38 h and the relative bioavailability was increased by nearly 20 times, indicating that less drug is needed for the same therapeutic effect by using the icariin complex, and the complex can be used as a potential potent hepatoprotector or anti-liver cancer drug.

## Introduction

**FIGURE 1:**
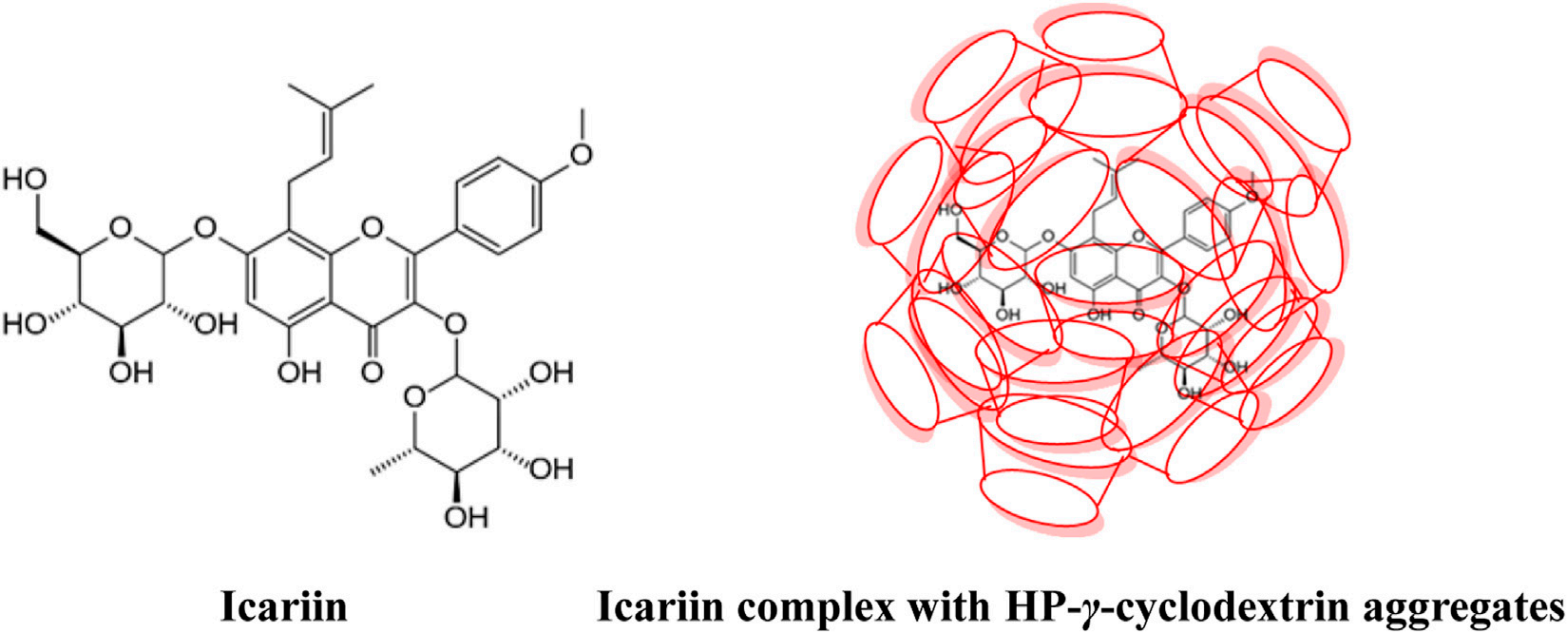
Structures of icariin and its complex with HP-*γ*-cyclodextrin aggregates.

Icariin is an isoamyl flavonol glycoside natural product (Figure 1) isolated from *Herba epimedii*. It can expand the cardiovascular and cerebral blood vessels, promote hematopoietic functions, enhance the immune system, promote the synthesis of protein and DNA, show anti-aging and anti-liver tumor activities, and improve the bone formation of osteoblasts (Shen and Wang, 2018). However, its low solubility (0.02 mg/mL) limits its clinical applications as food and medical supplements (Wang et al., 2013a).

When icariin was added to an aqueous solution of hydroxypropyl-*β*-cyclodextrin (40% W/V), after stirring at 40°C for 4 h, the solubility of icariin was increased 223 folds to 4.46 mg/mL. However, the ratio of icariin to HP-*β*-cyclodextrin is close to 1:100, which is impractical for clinical application (Chen and Huang, 2006). Through a freeze-drying process, the icariin/*β*-cyclodextrin inclusion complex was prepared and confirmed, but the drug release was improved only 5% compared with that of icariin alone (Zhang et al., 2009a). Its intestinal absorption mechanism in rats was studied (Zhang et al., 2012), when the complex was loaded into poly L-lactic acid scaffolds, the accumulated release percentage *in vitro* reached approximately 80% in 5 days (Zhang et al., 2009b). By using the dropping method, the inclusion complex of icariin with *β*-cyclodextrin was prepared, and the water solubility of icariin was increased only from 17 to 36 times though complexation (Jin et al., 2012; Cui et al., 2013). Complexation of HP-*β*-cyclodextrin with icariin by stirring can only increase icariin’s water solubility about 4.3 times (Wang et al., 2013b). Different concentrations of *β*-cyclodextrin, HP-*β*-cyclodextrin and random methylated-*β*-cyclodextrin were used to form inclusion complexes with icariin at different temperatures, and the water solubility of icariin was increased from 1.2 to 8.5 times (Wang et al., 2020). When *β*-cyclodextrin conjugated alginate was complexed with icariin, the inclusion nanocomplex achieved sustained icariin release for up to 7 days (Choi et al., 2020a), when nanodiamonds were functionalized with icariin, the icariin release can be prolonged for up to 4 days (Choi et al., 2020b).

Recently, nanotechnology has become very popular for application in drug delivery. Nanotechnology can improve the absorption and delivery of drugs with low water solubility through encapsulation in nanocarriers (Gunasekaran et al., 2014). The complexation of icariin with phospholipids did not change icariin’s water solubility, but increased its bioavailability (Pan et al., 2018). A mixture of self-assembled micelles and icariin increments achieved 500% bioavailability compared with that of icariin (Han et al., 2019). Fetal bovine serum was incorporated with icariin and improved the proliferation of cells compared to the plain icariin (Dong et al., 2021). Hyaluronic acid–icariin hydrogels can mitigate the burst release of icariin and achieve long-lasting bioactivity of icariin containing hydrogels (He et al., 2014). Gelatin/hyaluronic acid-icariin microspheres can achieve a slower release of icariin when the icariin level is lower (Yan et al., 2016).

Lyophilized icariin stealth solid lipid nanoparticles can reduce the mobility of icariin in the lipid, prevent coalescence and ensure continuous release of icariin (Liu et al., 2012). From two polymorphs in pure form and ten different solvate modifications of icariin, an α anhydrous form was found to show higher water solubility (Jia et al., 2015). These nanotechnology research results did not show significant icariin water solubility improvement and involved the use of polymers which could lead to problems in the production and quality control of the drug.

Overall, even although tremendous efforts have been made toward increasing the solubility of icariin, the results are not impressive, and it is necessary to develop a drug carrier to improve icariin’s water solubility. In continuation of interest in drug inclusion complex studies (Ding et al., 2018; Ding et al., 2020; Ding et al., 2021; Ding et al., 2022) with cyclodextrin derivatives, icariin’s water solubility can be increased more than 654 folds, and these results are reported in detail in this communication.

## Methods and materials

Ethyl acetate was purchased from Guangdong Guanghua Sci-tech Co., Ltd., anhydrous ethanol was purchased from Tianjin Fuyu Fine Chemical Co., Ltd, hydroxypropyl-*γ*-cyclodextrin and icariin (95% and 99%) were purchased from Shanghai Macklin, citric acid, HP-*β*-cyclodextrin and *β*-cyclodextrin were purchased from Sa’en Chemistry Technology, anhydrous sodium acetate was purchased from Xilong Science Co., Ltd, hydroxypropyl methyl cellulose, benzalkonium chloride, sodium hydroxymethyl cellulose and hexadimethrine bromide were purchased from Shanghai Maclean Biochemical Technology Co., Ltd, sodium alkyl (12 carbons) sulfate was purchased from Tianjin Best Chemical Co., Ltd, polyvinylpyrrolidone was purchased from Tianjin Tianxin Fine Chemical Development Center, polyethylene glycol 4000 was purchased from Tianjin Yongda Chemical Reagent Co., Ltd, sodium salicylate was purchased from Shanghai Lingfeng Chemical Reagent Co., Ltd, the supercritical carbon dioxide reactor BZ-100ML/S0-L was purchased from Baikal Shanghai Intelligent Tecchnology Co., Ltd, the autoclave was obtained from Digestion high-pressure tank Nanjing Ruinik Technology Development Co., Ltd, the microwave reactor WBFY-201 was purchased from Gongyi Yuhua Instrument Co., Ltd.

The UV spectra were recorded with an ultraviolet visible spectrophotometer, J51903001 from Shanghai Jinghua Technology Instrument Co., Ltd. The ^1^H NMR spectra were recorded on a Bruker spectrometer (400 MHz) by using D_2_O or DMSOd_6_ as solvent. The HPLC analysis was performed on a LC-15C high performance liquid chromatograph from Shimadzu Enterprise Management (China) Co., Ltd. By using an Inertsil ODS-3 C18 (250 mm × 4.6 mm) column. The dissolution testing was performed on an RC-3 dissolution meter from Tianjin Xintianguang Analytical Instrument Technology Co., Ltd. The X-ray analysis was performed on an X-ray polycrystalline diffractometer, D8 ADVANCE from Bruker. The FTIR spectra were recorded on a VERTEX 70 Fourier transform infrared spectrometer from Bruker. The scanning electron microscopic images were recorded on a Zeiss MERLIN high resolution field emission scanning electron microscope. Thermogravimetric analysis was finished on Mettler Toledo TGA2 from NETZSCH GmbH and Co. Holding KG (ENH). The differential scanning calorimetry was recorded on a DSC 214 differential scanning calorimeter from Netzsch, Germany.

Dogs were obtained from the Guangdong Medical Laboratory Animal Center, and the blank dog plasma was obtained from Guangzhou Rui-Te Co., Ltd. Disposable blood collection needles and 5 mL anticoagulation tubes were obtained from Shenzhen Kangzhijian Medical Equipment Biotechnology Co., Ltd, 2 mL centrifuge tubes were obtained from BIOFIL. Clean centrifuge tubes and 0.22 *μ*m microporous organic filters were obtained from Tianjin Jinteng Experimental Equipment Co., Ltd. Nitrogen concentrators, NDK200-1N, were obtained from Hangzhou Miou Instrument Co., Ltd.

### Determination of icariin’s maximum absorbance UV wavelength

A solution of HP-*γ*-cyclodextrin in water (1 mg/mL, 1 mL) and a solution of icariin in methanol (1 mL, 1 mg/mL) were filtered and transferred to disposable quartz cuvettes. Their UV spectra were recorded at wavelengths from 200 nm to 400 nm. The icariin solution showed the maximum absorbance at 270 nm, which was selected as the wavelength for HPLC analysis, since cyclodextrin solution did not show absorption in this range.

### Icariin HPLC standard curve establishment

The solution of icariin (1 mg) in methanol (5 mL) was diluted with methanol to give a series of samples with 0.01 mg/mL, 0.02 mg/mL, 0.04 mg/mL, 0.06 mg/mL and 0.08 mg/mL concentrations for HPLC analysis. The wavelength in HPLC was set at 270 nm, a Shim-packVP-ODS C18 column (250L×4.6 mm) was used for analysis at 30°C, and the mobile phase was acetonitrile: water (30:70, V/V) with a flow rate of 1.0 mL/min. Based on the areas of HPLC peaks and the concentrations in the range of 20 *μ*g/mL–100 *μ*g/mL, a linear regression equation was obtained as Y = 21969X-41.232 (coefficient of determination *R* ^2^ = 0.9992, *Y* = absorption peak area, *X* = concentration).

### Phase solubility analysis

Solutions of *β*-cyclodextrin, HP-*β*-cyclodextrin, methyl-*β*-cyclodextrin, citric acid-*β*-cyclodextrin, *γ*-cyclodextrin and HP-*γ*-cyclodextrin in water with 100, 110, 120, 130, 140 and 150 mg/mL concentrations were prepared, 10 mg icariin was added to each solution, the mixture was stirred at low speed at room temperature for 48 h, 1 mL of each reaction mixture was filtered and analyzed by HPLC.

### Preparation of the inclusion complex of icariin with HP-*γ*-cyclodextrin

**Solution method**: the solution of icariin (100 mg) in anhydrous ethanol (100 mL) was added dropwise to the solution of HP-*γ*-cyclodextrin (235 mg) in ultrapure water (1 mL). The resulting solution was stirred at 60°C at 1000 rpm for 12 h, the ethanol was evaporated under reduced pressure, ultrapure water (5 mL) was added, after filtration, the solution was lyophilized to give the product.

**Sonication method**: the solution of icariin (100 mg) in anhydrous ethanol (100 mL) was ultrasonicated at 25°C and 300 W, followed by slowly addition of the solution of HP-*γ*-cyclodextrin (235 mg) in ultrapure water (1 mL). After 30 min, the solution was evaporated to remove the ethanol, added ultrapure water (5 mL), filtered and lyophilized to give the product.

**Using autoclave**: the solution of icariin (100 mg) and HP-*γ*-cyclodextrin (235 mg) in the mixture of ethanol and water (1:1, 80 mL) was stirred at 120°C and 800 rpm for 8 h. After removal of ethanol through evaporation, ultrapure water (5 mL) was added, after filtration and lyophilization, the product was obtained as a white powder.

**Using microwave reactor**: the solution of icariin (100 mg) and HP-*γ*-cyclodextrin (235 mg) in the mixture of ethanol and water (100:1, 80 mL) in the microwave reactor was stirred at 500 rpm, and every 30 s the reaction was stopped for 1 min. After 30 min, the mixture was evaporated to remove the ethanol, washed with water, filtered and lyophilized to give the complex.

**Using supercritical CO**_**2**_: the solution of icariin (100 mg) in ethanol (60 mL) was added to the solution of HP-*γ*-cyclodextrin (235 mg) in 1 mL of water in a reaction kettle. Liquid CO_2_ was added to keep the pressure as 8.0 Mpa, and then the mixture was stirred at 50°C for 5 h and at 70°C for 5 h. After releasing the pressure at room temperature, the mixture was evaporated to remove the ethanol, filtered and freeze-dried to give the product.

**Preparation of the physical mixture of icariin and HP-*γ*-cyclodextrin**: icariin and HP-*γ*-cyclodextrin (100 mg, 235 mg) were mixed, grounded and passed through 80 mesh sieve to get the physical mixture of icariin and HP-*γ*-cyclodextrin.

### Characterization of the complex

**Fourier transform infrared spectroscopy study**: FTIR spectra of the KBr pellets of icariin, HP-*γ*-cyclodextrin, the physical mixture of icariin and HP-*γ*-cyclodextrin, and the inclusion complex were analyzed by Fourier transform infrared spectroscopy, and the requisite data were collected between 500 cm^-1^ and 4500 cm^-1^.

**Scanning electron microscopy study**: the microstructure and surface morphology of icariin, HP-*γ*-cyclodextrin, the physical mixture of icariin and HP-*γ*-cyclodextrin, and their inclusion complex were studied by using a scanning electron microscope at an accelerating voltage of 5 kV under high vacuum. Each solid sample was placed on the magnetic block and, after gold spray treatment, the sample was loaded onto the sample rod for scanning. The image magnification was 500 times, and the image type was secondary electron. The scale bar of the images is 10 *μ*m.

**Thermogravimetric Analysis:** icariin, HP-*γ*-cyclodextrin, the physical mixture of icariin and HP-*γ*-cyclodextrin, and the inclusion complex were analyzed by thermogravimetry in a nitrogen atmosphere. The experiments were performed under the following conditions, flow rate: 10 mL/min, temperature range: 30°C–300°C, heating rate: 10°C/min.

**Differential scanning calorimetry study**: the curves of icariin, HP-*γ*-cyclodextrin, the physical mixture of icariin and *γ*-cyclodextrin, and their inclusion compound were obtained by placing in an aluminum crucible and heating at a rate of 10°C/min From 70°C to 350°C under nitrogen atmosphere with 150 mL/min Flow rate.

**X-ray diffraction analysis**: icariin, HP-*γ*-cyclodextrin, their physical mixture and inclusion complex were analyzed by X-ray diffraction using Cu-k α emission with a LYNXEYE detector, with a voltage of 40 kV and a current of 40 mA. The scanning angle range was 3–60° and the scanning speed was 0.01/*s*.

**Proton NMR study**: the solutions of HP-*γ*-cyclodextrin and its inclusion complex with icariin in D_2_O, the solutions of griseofulvin and its inclusion complex in DMSO-d_6_ had their 1D NMR spectra recorded at 400 MHz at room temperature.

**Determination of the icariin content in the complex**: the inclusion complex (100 mg) was dissolved in deionized water (1 mL), the solution was diluted to the range of 8 *μ*g/mL–500 *μ*g/mL, based on HPLC analysis, and the icariin content in the complex was obtained through the regression equation.

**Determination of water solubility of icariin in complex**: the inclusion complex of icariin was added to water (1 mL) to get its saturated aqueous solution. After filtration, the solution was analyzed by HPLC to get the water solubility of icariin in the complex.

**Determination of the inclusion rate and inclusion yield**: the inclusion rate and yield of icariin inclusion compound can be obtained based on following formulae:

Inclusion yield (%) = [icariin inclusion complex (mg)/icariin (mg) + HP-*γ*-cyclodextrin (mg)] x 100%; Inclusion ratio (%) = [icariin in inclusion complex (mg)/icariin (mg)] x 100%.

**Dissolution rate determination**: the solution of icariin (100 mg), the physical mixture of icariin (100 mg) and HP-*γ*-cyclodextrin (235 mg), and the inclusion complex (containing 100 mg of icariin) in 900 mL of water were each ultrasonically vibrated at 37°C with stirring (100 rpm). A 1 mL sample was taken from each of the solutions at 2, 5, 10, 15, 20, 30, 45 and 60 min, followed by the addition of 1 mL of ultrapure water at 37°C at the same times. After filtration, the samples were analyzed by HPLC and, based on the HPLC data, the *in vitro* dissolution rate was obtained.

### *In vivo* pharmacokinetic studies

**Sample preparation:** twelve healthy adult dogs of around 5 ± 0.1 kg, half male and half female, were randomly divided into two groups designated as drug group and inclusion group. Before the experiments, the dogs were fed and kept in a warm and ventilated environment. Then, after fasting for 1 day, the dogs were orally dosed with drug and drug inclusion complex (20 mg/mL). During the experiments, the dogs were fed with low-fat food and normal drinking water. Blood samples (2 mL) were collected through the anterior vena cava at 0, 0.08, 0.25, 0.5, 0.75, 1, 2, 4, 6, 8, 12, 24 and 48 h after administration of the drugs with a negative pressure disposable blood collection tube containing heparin sodium. The samples were transferred to a centrifuge tube and centrifugated at 4000 *r*/min for 10 min. The supernatant plasma was numbered and stored at −20°C. The plasma samples were thawed to room temperature, 0.5 mL of it was pipetted into a 2 mL centrifuge tube, 1 mL of analytical ethyl acetate was added and, after vortexing for 5 min, the sample was centrifuged at 13000 r/min for 10 min. The supernatant was transferred to a test tube, the procedure was repeated three times, and the collected solution was blown with nitrogen at 40°C. The residue was dissolved in 0.5 mL methanol, vortexed for 5 min, centrifuged at 13000 *r*/min for 10 min, filtered through a 0.22 *μ*m microporous membrane and analyzed by HPLC.

**Standard curve for icariin in plasma**: the solution of 10 mg of icariin in 2 mL of methanol was diluted in a 10 mL volumetric flask with methanol, sonicated to provide a solution with 1 mg/mL concentration, which was diluted with the mobile phase to get a solution of 100 *μ*g/mL concentration, pipetted with 0.1 mL of this solution into a 2 mL centrifuge tube, added 0.4 mL of blank plasma and vortexed for 5 min. The resulting plasma solution of 20 *μ*g/mL concentration was diluted to a series of samples with different concentrations for HPLC analysis. The HPLC analysis was performed at a wavelength of 270 nm using a Shim-pack VP-ODS C18 column (250 L×4.6 mm) with a mobile phase of acetonitrile: water (30:70, V/V) at 30°C with flow rate of 1.0 mL/min and injection volume of 10 *μ*L. Based on the peak areas in the HPLC results and the concentrations of the samples, the standard curve for icariin in plasma was obtained.

**Statistical analysis:** analyses were carried out in triplicate. All results were subjected to statistical analysis to obtain average values and a one-way analysis of variance ANOVA using STAT- ISTICA 10 software (StatSoft Inc., Tulsa, United States) at a significance level of *p* ≤ 0.05.

## Results and discussion

**FIGURE 2:**
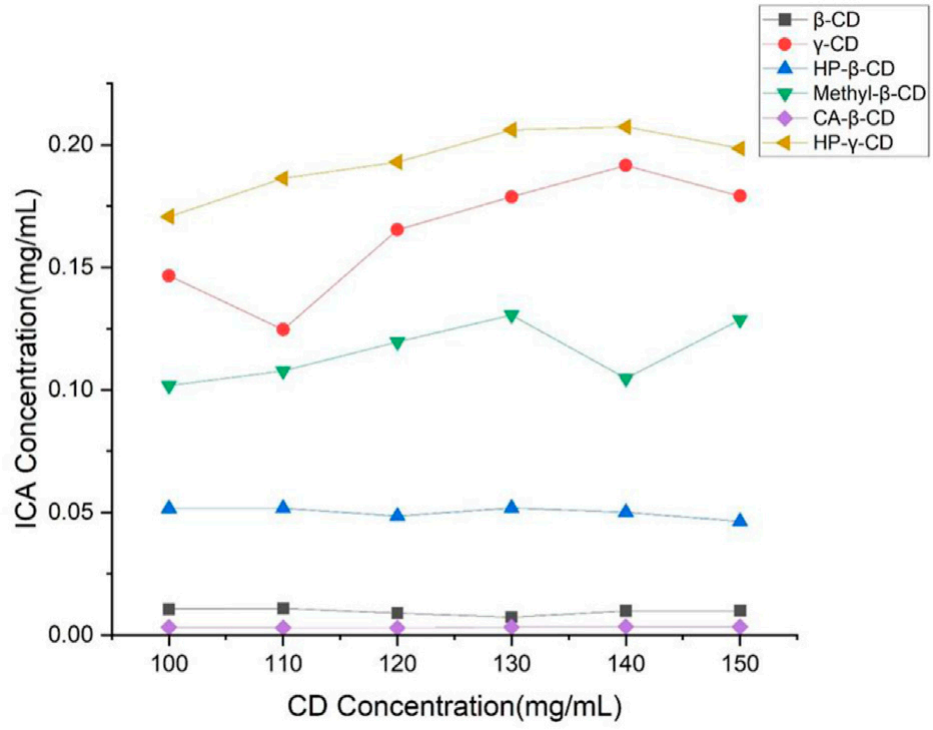
Phase solubility curves of icariin with *β*-cyclodextrin, HP-*β*-cyclodextrin, methyl-*β*-cyclodextrin, citric acid-*β*-cyclodextrin, *γ*-cyclodextrin and HP-*γ*-cyclodextrin.

*β*-Cyclodextrin, HP-*β*-cyclodextrin, methyl-*β*-cyclodextrin, *γ*-cyclodextrin and HP-*γ*-cyclodextrin as commercial products are most commonly used as drug carriers in the literature. Also, citric acid-*β*-cyclodextrin was available, therefore, the solutions of these cyclodextrins in different concentrations were treated with icariin for 48 h with stirring, and samples of these solutions were filtered and analyzed by HPLC. The solubility of icariin is shown in Figure 2. Under the same concentrations, HP-*γ*-cyclodextrin was found to have the best solubilization effect on icariin. According to the Higuchi-Connors equation, icariin’s phase solubility curve can be classified as A_N_ type.

There are many methods to prepare the inclusion complex such as aqueous solution, ultrasonic, microwave reactor, autoclave reactor and supercritical CO_2_ assistance. Each method was used to form the complex of icariin with HP-*γ-*cyclodextrin. The results were summarized in Table 1.

**TABLE 1:** The water solubilities of icariin in the complex from different methods.

Methods	Solubility
Solution method	1.3 mg/mL
Sonication method	0.8 mg/mL
Using microwave reactor	1.2 mg/mL
Using autoclave	0.96 mg/mL
Using supercritical CO_2_	1.1 mg/mL

The complex obtained by the aqueous solution method showed the solubility of icariin to be 1.3 mg/mL based on HPLC analysis, the ultrasonic method gave the result as 0.8 mg/mL, the microwave reactor gave 1.2 mg/mL water solubility, the autoclave reactor gave the solubility as 0.96 mg/mL, and the supercritical CO_2_ assisted reaction gave the solubility as 1.1 mg/mL. Therefore, aqueous solution method was selected to form the complex with HP-*γ*-cyclodextrin.

Accordingly, the complexation conditions between icariin and HP-*γ*-cyclodextrin by using solution methods were optimized through a single factor strategy. The solution of icariin in ethanol was dropped into the solution of HP-*γ*-cyclodextrin in water (molar ratio: 1:1) slowly, and the mixture was stirred at 40°C, 50°C or 60°C for 6 h with 1000 RPM stirring speed. The complex from the reaction at 50°C gave a better icariin water solubility as 1.6 mg/mL than the others. Then, the solution of icariin in ethanol was slowly dropped into the solution of HP-*γ*-cyclodextrin in water with 1:1 M ratio, stirred at 50°C at 1000 rpm for 6, 8, 10 or 12 h. The reaction condition with 10 h stirring gave the best solubility increasing to 6.84 mg/mL. The complexation solutions with 1:1, 1:2 and 1:3 M ratio between icariin and HP-*γ*-cyclodextrin were stirred at 1000 rpm at 50°C for 10 h, and it was found that the ratio of 1:1 gave the best result as 7.83 mg/mL.

It is known that cyclodextrins and its complexes can self-associate to form aggregates or micelle-like structures to solubilize lipophilic water-insoluble drugs through non-inclusion complexation (Loftsson et al., 2019). Larger aggregates will limit their water solubilization capability (Karandur et al., 2014), and water-soluble polymer surfactants and anionic and cationic molecules can be used to stabilize all kinds of aggregates (Khan and Brettmann, 2019). HP-*γ*-cyclodextrin may form both inclusion and non-inclusion complexes with icariin and coexist in aqueous solutions. Small amounts (0.25%) of water-soluble polymers and organic salts such as sodium salicylate, hydroxypropyl methylcellulose, sodium carboxymethyl cellulose, polyvinylpyrrolidone, polyethylene glycol 4000, sodium dodecyl sulfate, hexadimethrine bromide, tris(hydroxymethyl)aminomethane, sodium acetate or benzalkonium chloride were added to the complexation reaction. After work up and HPLC analysis, it was found that the complexation with addition of sodium dodecyl sulfate could increase icariin’s water solubility to 13.09 mg/mL, which is the best result obtained so far and is 654 times higher than that of the icariin (Szabó et al., 2022). The inclusion rate and yield of icariin inclusion compound were obtained as 1.8% and 78.12% respectively, and the product was used for inclusion complex confirmation and *in vitro* and *in vivo* pharmacokinetic studies.

**FIGURE 3:**
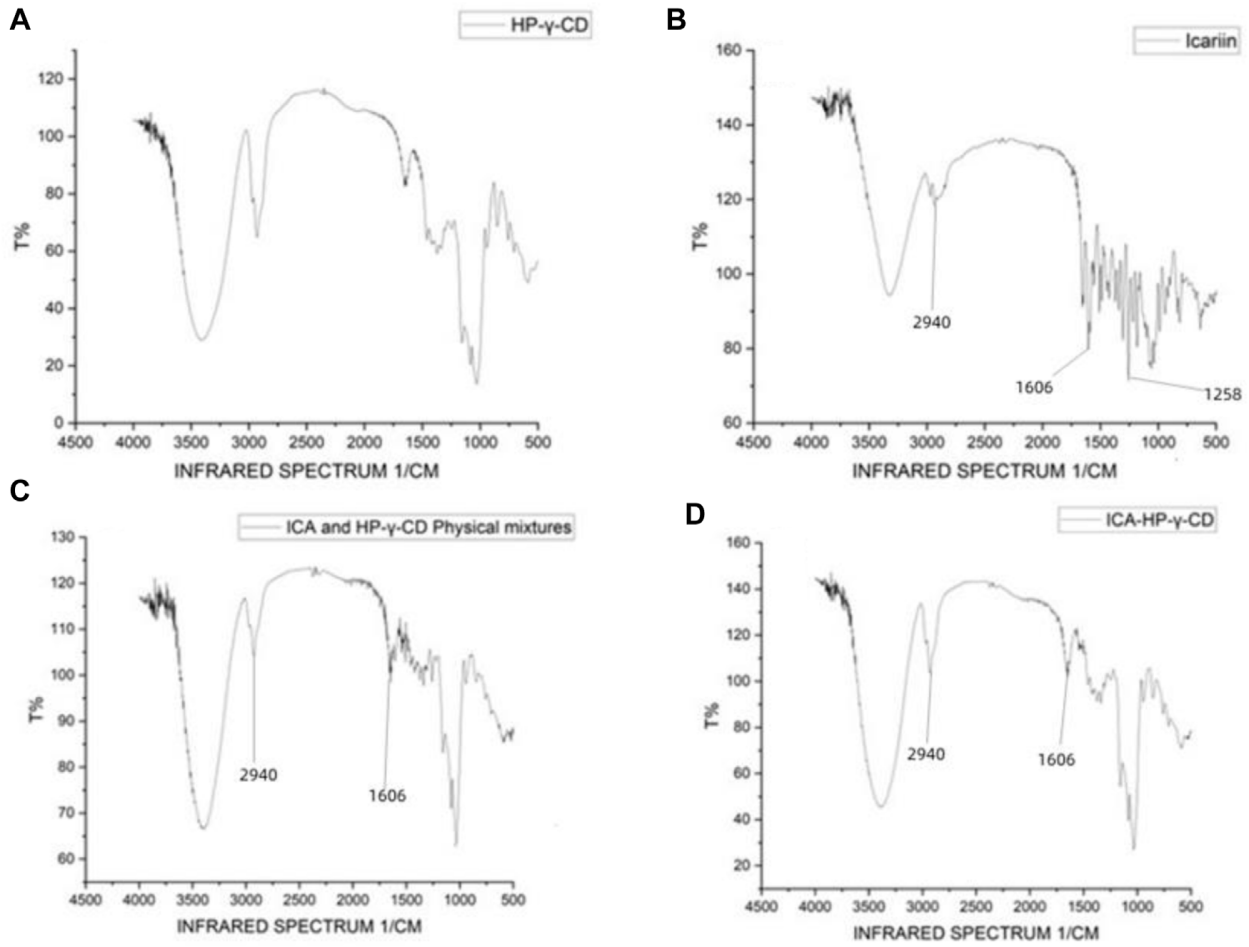
Fourier-transform infrared spectroscopy spectra of HP-*γ*-cyclodextrin **(A)**, icariin **(B)**, the physical mixture of HP-*γ*-cyclodextrin and icariin **(C)**, and their inclusion complex **(D)**.

FTIR spectra were used to confirm the formation of the complex between icariin and HP-*γ*- cyclodextrin (Figure 3). Comparing with the spectrum of HP-*γ*-cyclodextrin (1a), the characteristic peaks of icariin appeared at 2940, 1606 and 1258 cm^−1^ (1b) corresponding to methylene (CH_2_), benzene ring and methoxy group (OCH_3_) respectively, and they are obviously seen in the physical 5mixture of icariin and HP-*γ*-cyclodextrin (1c), but have slightly changed and reduced in the inclusion complex (1d). These changes of characteristic peaks of icariin confirmed that the icariin existed as an inclusion complex with cyclodextrin.

**FIGURE 4:**
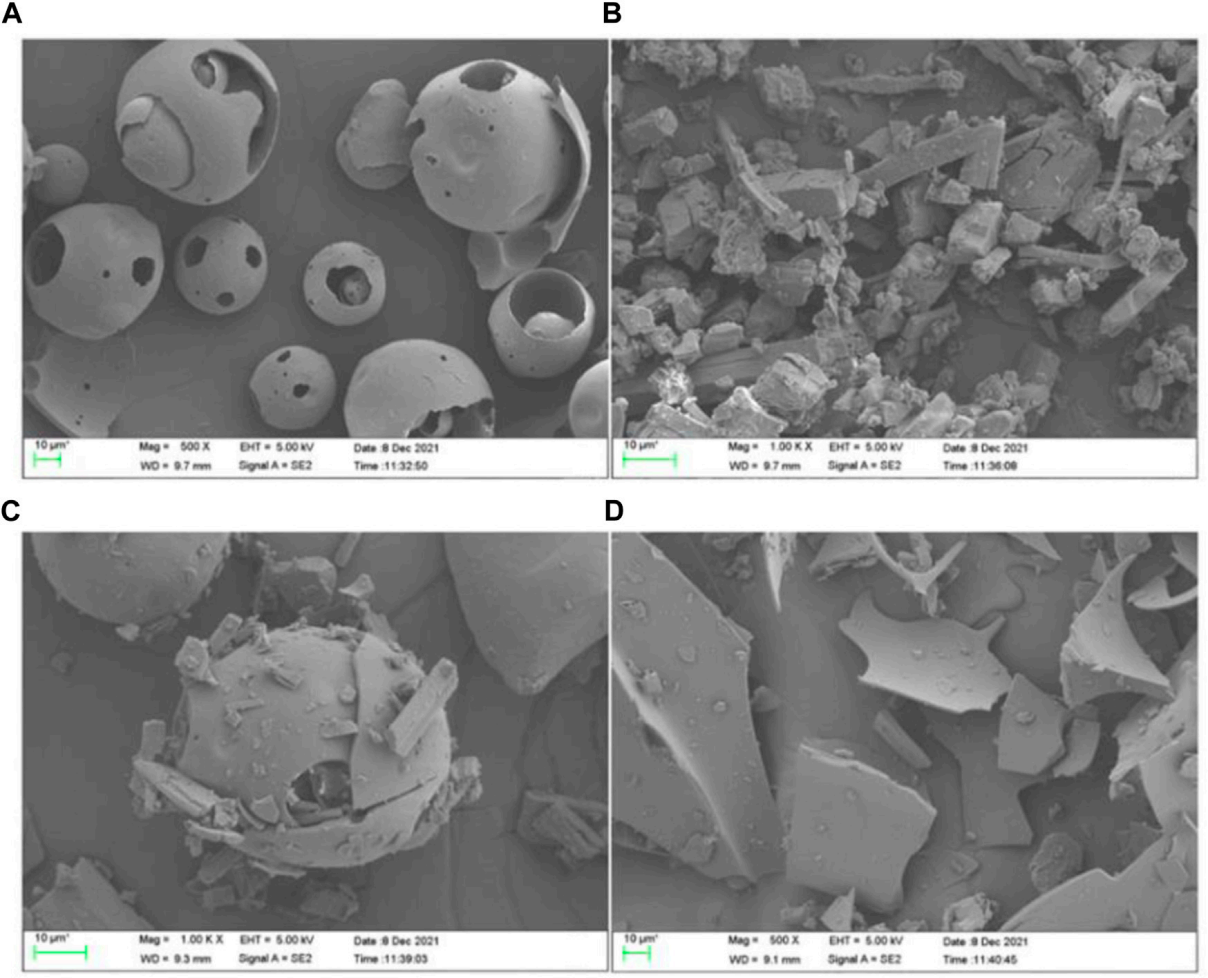
The scanning electron microscope images of HP-*γ*-cyclodextrin **(A)**, icariin **(B)**, the physical mixture of icariin and HP-*γ*-cyclodextrin **(C)**, and their inclusion complex **(D)**.

Scanning electron microscopy was used to study the microscopic aspects of the materials obtained by co-precipitation or evaporation, and can provide images to intuitively analyze whether the icariin inclusion complex is formed by comparing the image differences between HP-*γ*-cyclodextrin and the complex. They can also be used to demonstrate the changes in crystal states and spatial structures. As shown in Figure 4, HP-*γ*-cyclodextrin (A) showed spherical shapes of different sizes, icariin (B) presented block shapes, while the physical mixture (C) was a simple mixed accumulation of HP-*γ*-cyclodextrin and icariin. The inclusion complex (D) showed irregular fragmentations, which was different from A, B and C, and indicated that a new phase was generated.

**FIGURE 5:**
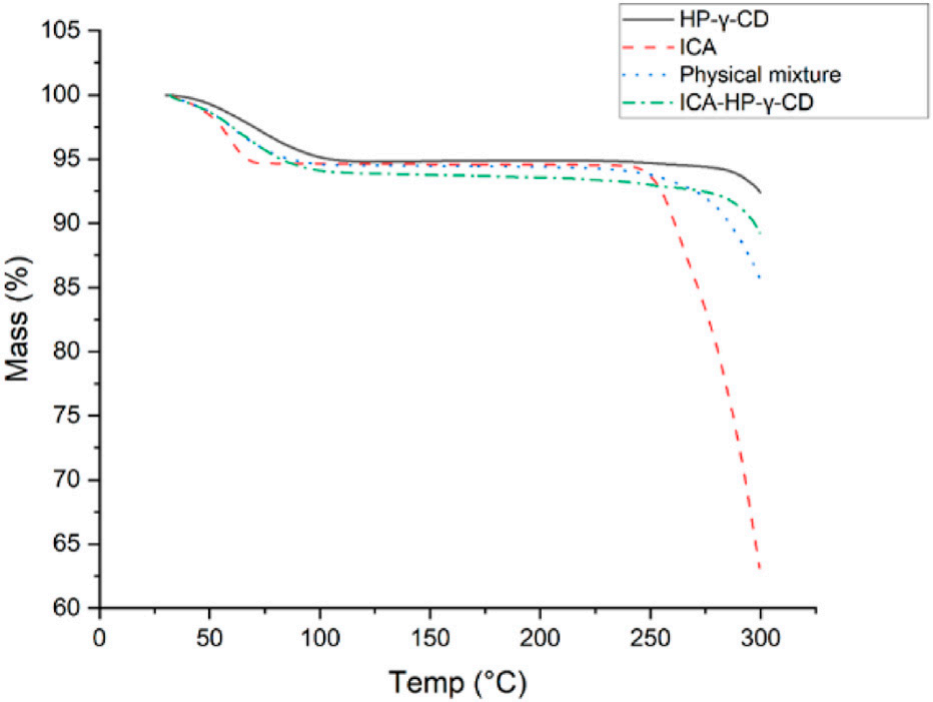
The thermogravimetric analysis of icariin, HP-*γ*-cyclodextrin, their physical mixture and inclusion complex.

The thermogravimetric analyses of icariin, HP-*γ*-cyclodextrin, their physical mixture and inclusion complex are summarized in Figure 5. It can be seen from the diagrams that the initial weight losses before 100°C were due to water evaporation in all the samples, and a second mass loses occurred between 250°C and 350°C due to the degradation of icariin. The order of their degradation temperatures was HP-*γ*-cyclodextrin > complex > mixture > icariin. Since the icariin gets protection from HP-*γ*-cyclodextrin in the complex, the complex is more stable than the physical mixture and the pure icariin, which indirectly confirms the formation of the complex of icariin with HP-*γ*-cyclodextrin.

**FIGURE 6:**
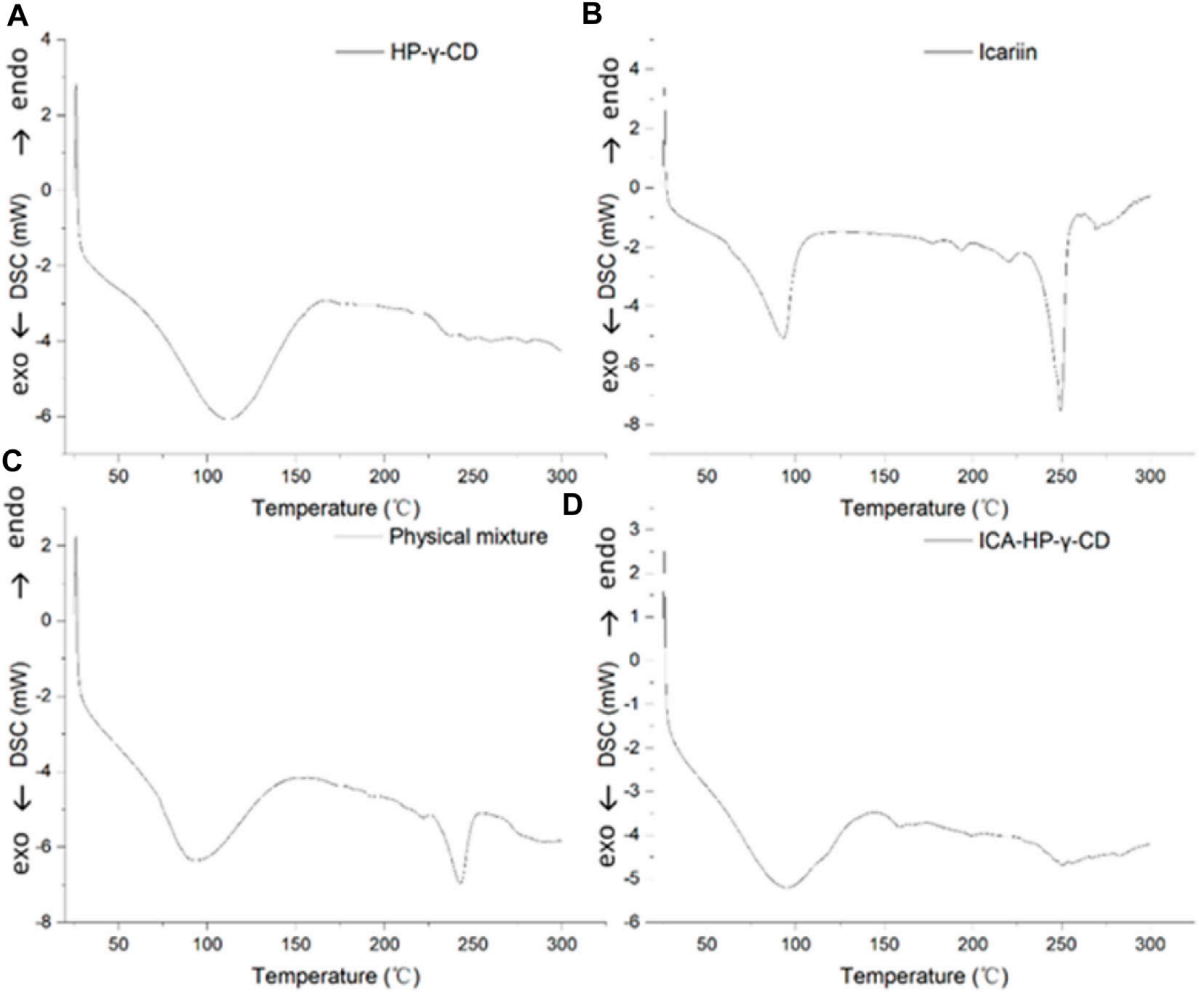
Differential scanning calorimetry curves of HP-*γ*-cyclodextrin **(A)**, icariin **(B)**, the physical mixture of HP-*γ*-cyclodextrin and icariin **(C)**, and their inclusion complex **(D)**.

The differential scanning calorimetry curves of icariin, HP-*γ*-cyclodextrin, their physical mixture and inclusion complex are shown in Figure 6. The curves present the endothermic events corresponding to dehydration. HP-*γ*-cyclodextrin has the thermal effects in the range of 75°C–150°C, and the endothermic peak at 110°C is attributed to evaporation of water molecules from the cavity of HP-*γ*-cyclodextrin. Icariin has a weak thermal effect in the range of 75°C–100°C due to the loss of crystal water, the endothermic peak at 250°C is assigned to its melting effect, the curve of physical mixture of HP-*γ*-cyclodextrin and icariin is the sum of HP-*γ*-cyclodextrin dehydration and the melting peak of icariin, while the inclusion complex has thermal effects in the range of 75°C–130°C. The characteristic endothermic peak of icariin at 250°C disappeared due to the loss of the crystalline structure caused by encapsulation, indicating that the interaction between icariin and HP-*γ*-cyclodextrin results in the formation of a new phase.

**FIGURE 7:**
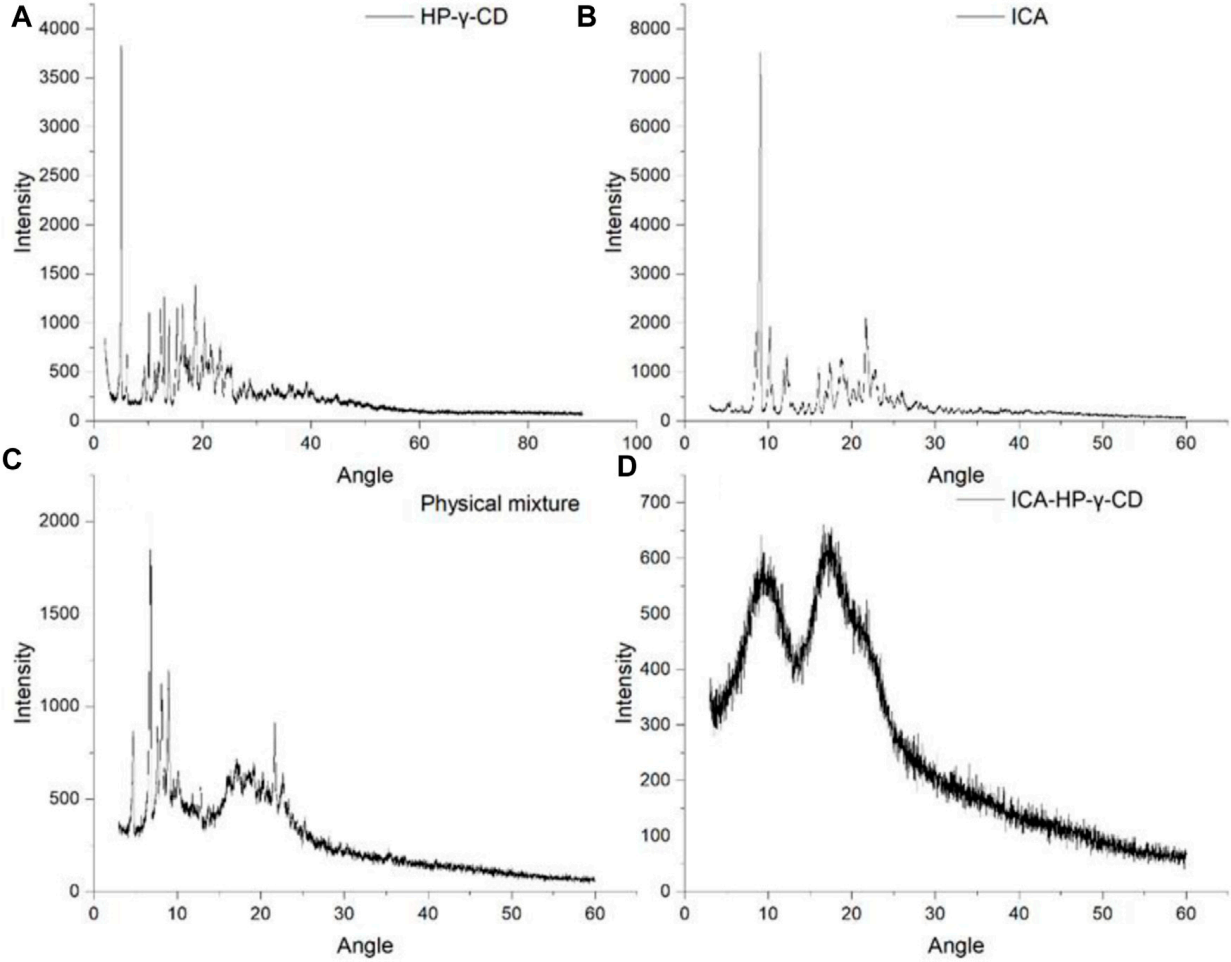
x-Ray diffraction patterns of HP-*γ*-cyclodextrin **(A)**, icariin **(B)**, the physical mixture of icariin and HP-*γ*-cyclodextrin **(C)**, and their inclusion complex **(D)**.

The X-ray diffractograms of icariin, HP-*γ*-cyclodextrin, their physical mixture and inclusion complex are shown in Figure 7. The crystalline solid patterns of the pure icariin (A) and HP-*γ*-cyclodextrin (B) were found in the spectra, and the peak shapes of the physical mixture (C) appear as the superposition of HP-*γ*-cyclodextrin and icariin. In the diffraction pattern of the inclusion complex (D), the characteristic peaks of HP-*γ*-cyclodextrin and icariin have disappeared completely, and an amorphous pattern is presented, indicating a new phase was formed. Thermal analysis findings are reinforced by results obtained by these x-ray measurements. All the changes observed and discussed in FTIR, TAG, DSC, X-ray and change in the morphology in SEM confirm the complex formation which is necessary for increasing solubility.

**FIGURE 8:**
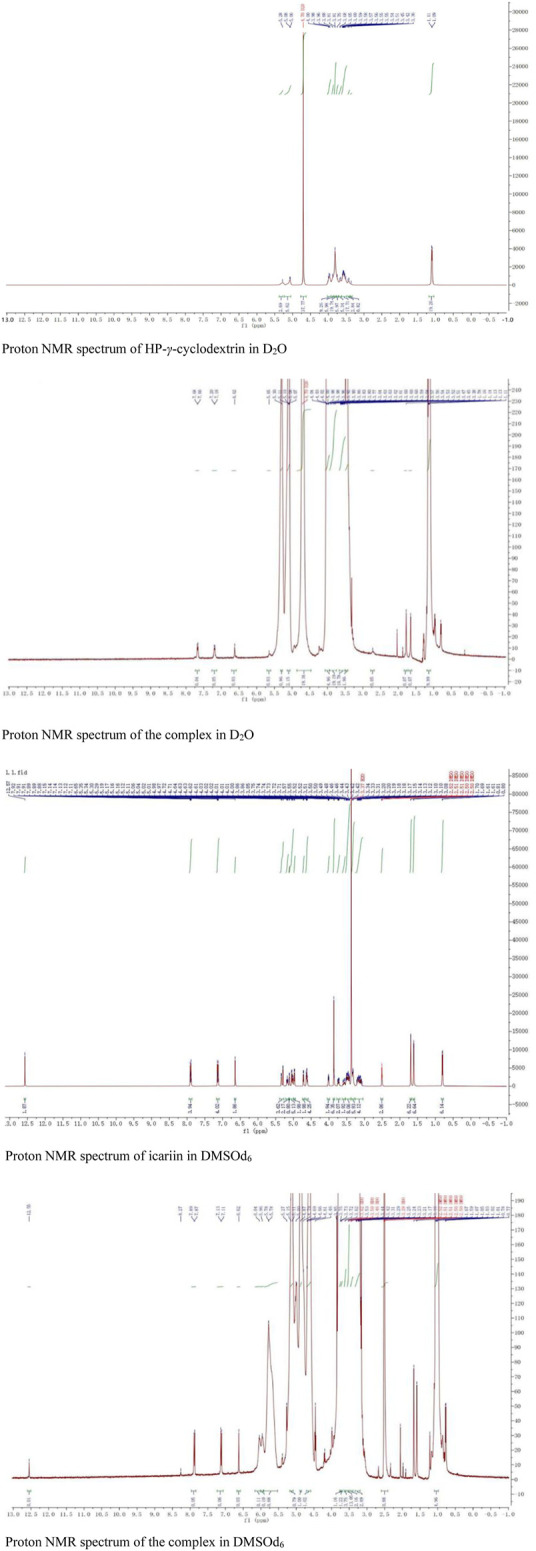
The proton NMR spectra of HP-*γ*-cyclodextrin and the complex in D_2_O, the proton NMR spectra of icariin and the complex in DMSOd_6_.

The proton NMR spectra of icariin in DMSOd_6_, HP-*γ*-cyclodextrin in D_2_O, and the inclusion complex in D_2_O and DMSOd_6_ are shown in Figure 8. The proton NMR spectrum of the complex in D_2_O exhibits the signals from icariin at 7.67 (2H, d, *J*_1_ = 8.0 Hz) (1 or 2), 7.19 (2H, d, *J*_1_ = 8.0 Hz) (1 or 2), 6.62 (1H, *s*) (3), 5.65 (1H, *s*) (4), 1.77 (3H, s) (7), 1.61 (3H, s) (7), 0.77 (3H, d, *J*_1_ = 4 Hz) (8); the signals of OCH_3_ (5) were overlapped with the signals from HP-*γ*-cyclodextrin in the range of 4.00 ppm to 3.50 ppm. The proton NMR spectrum of icariin in DMSOd_6_ shows the signals at 12.57 (1H, s), 7.90 (3H, d, *J*_1_ = 8.0 Hz), 7.13 (3H, d, *J*_1_ = 8.0 Hz), 6.65 (1H, s), 5.29 (1H, s), 3.85 (3H, s, OCH_3_), 1.70 (3H, s), 1.61 (3H, s), 0.81 (3H, d, *J*_1_ = 4.0 Hz); the proton NMR data of icariin in the complex in DMSOd_6_ shows the signals at 12.56 (1H, s), 7.88 (2H, d, *J*_1_ = 8.0 Hz), 7.12 (2H, d, *J*_1_ = 8.0 Hz), 6.62 (1H, s), 1.67 (3H, s), 1.59 (3H, s), 0.77 (3H, d, *J*_1_ = 4.0 Hz). All the proton chemical shifts of icariin in the complex undergo a shift (0.01 ppm–0.02 ppm) to the up-field region compared with that of icariin alone, indicating the external interaction between the icariin and HP-*γ*-cyclodextrin. The icariin cannot enter the inner cavity of HP-*γ*-cyclodextrin and interacts with the outer segment of the cavity, probably forming the complex with HP-*γ*-cyclodextrin aggregates. It is the first time that the proton NMR of the complex in D_2_O was used to confirm the water solubility of icariin in the complex with cyclodextrins.

**FIGURE 9:**
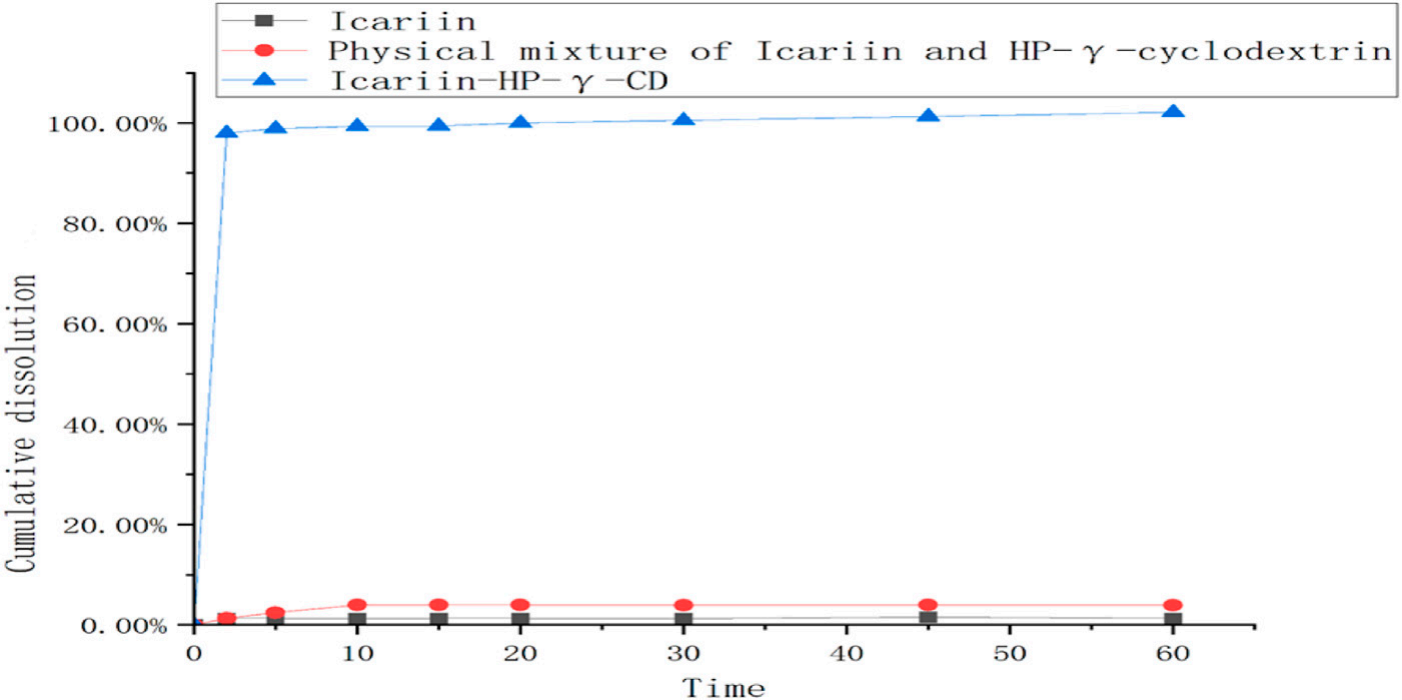
The dissolution curves of icariin, the mixture of icariin and HP-γ-cyclodextrin, and their complex.

The dissolution rates of icariin, the physical mixture of icariin and HP-*γ*-cyclodextrin, and their complex are summarized in Figure 9. The icariin in the complex can be 100% released during the first 10 min, which is about 80 times better than that of icariin alone, and 20 times better than its physical mixture with HP-*γ*-cyclodextrin.

**FIGURE 10:**
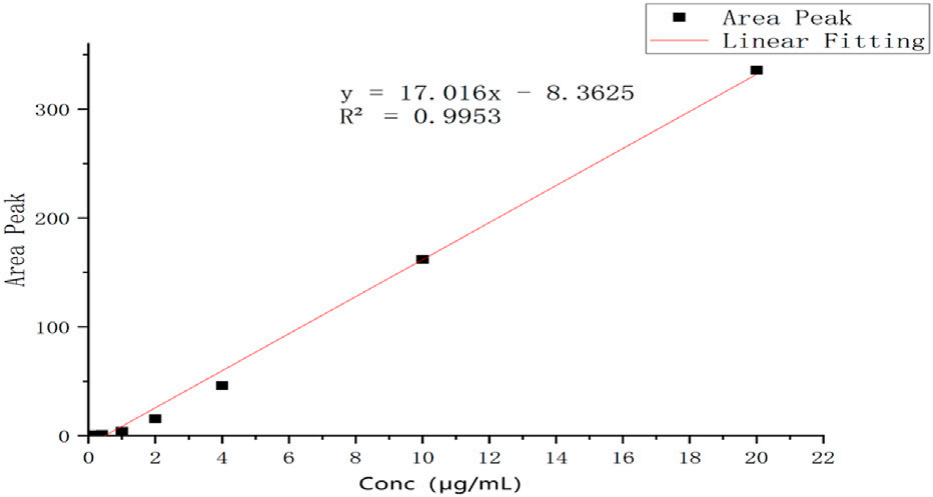
The standard curve of icariin in blank dog plasma.

A series of solutions of icariin in blank plasma with different concentrations were analyzed by HPLC in the range of 0.2 *μ*g/mL to 20 *μ*g/mL. The peak areas in HPLC and the concentrations of icariin in plasma solutions have a linear relationship, and the standard curve equation was obtained as: *Y* = 17.016X −8.3625 (coefficient of determination *R* ^2^ = 0.9953, Y = peak area, X = concentration, S/N ≥ 3: LLOD = 0.2 *μ*g/mL, S/N ≥ 10, LLOQ = 0.5 *μ*g/mL). The standard curve is shown in Figure 10.

**FIGURE 11:**
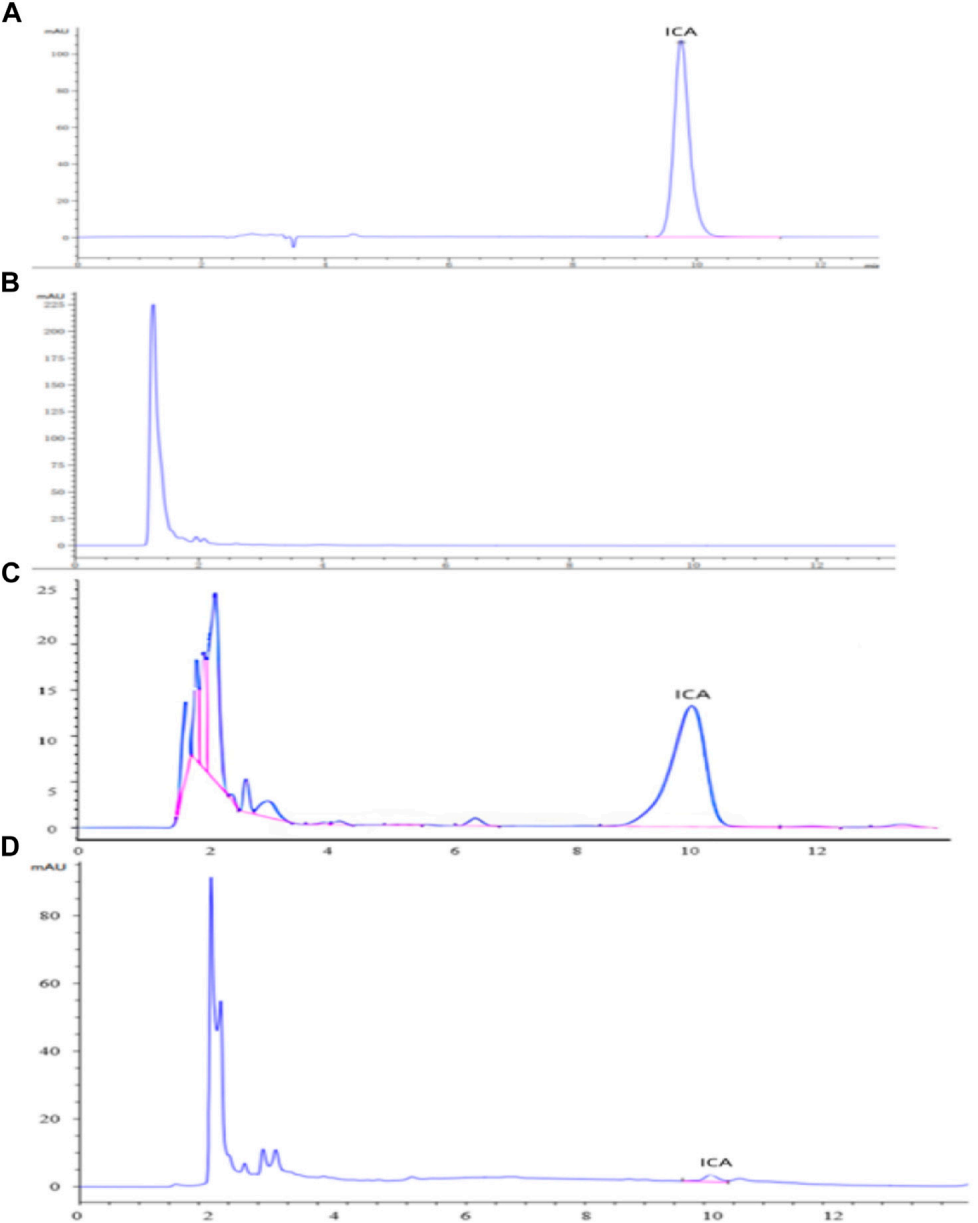
HPLC analysis of icariin **(A)**, blank dog plasma **(B)**, plasma containing icariin **(C)**, and the plasma from the dogs dosed with icariin/*γ*-cyclodextrin complex **(D)**.

The HPLC analyses of icariin (**A**), the blank dog plasma (**B**), the dog plasma containing icariin (**C**), and the plasma from the dogs dosed with icariin/HP-*γ*-cyclodextrin complex (**D**) are shown in Figure 11. The blank dog plasma did not interfere with the detection of icariin.

The recovery rate, intra-assay coefficient of variation, and the inter-assay coefficient of variation of icariin standard solution in blank plasma with concentrations of 1 *μ*g/mL, 10 *μ*g/m and 20 *μ*g/mL were determined. The recovery rate of icariin in plasma is in the range of 99.48% ± 0.46% to 108.75% ± 6.1%, the intra-assay coefficient of variation is in the range of 0.40%–2.46%, and the inter-assay coefficient of variation is in the range of 1.02%–3.56%.

**FIGURE 12:**
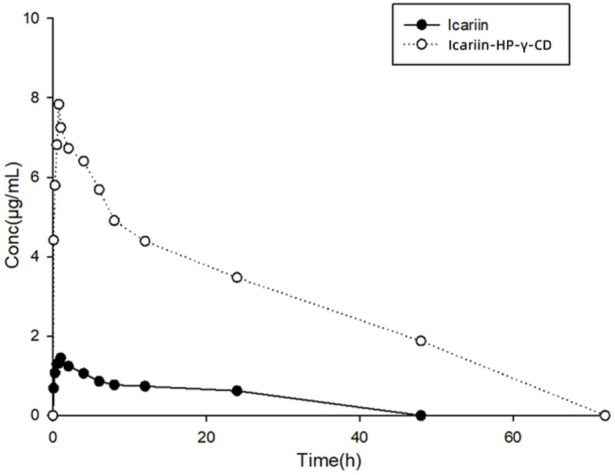
Curves of drug concentrations in plasma from dogs administered with icariin or its HP-*γ*-cyclodextrin complex.

The collected plasma samples from dogs dosed with icariin or its complex with HP-*γ*-cyclodextrin were analyzed by HPLC, and the results are shown in Figure 12. Pharmacokinetic parameters were calculated using Phoenix WinNonlin software. The relationship between the icariin concentration in dog plasma and time conforms the first-level absorption two-compartment model, and the pharmacokinetic parameters (mean ± SD) are summarized in Table 2.

**TABLE 2:** Pharmacokinetic parameters of icariin and its HP-*γ*-cyclodextrin complex.

Parameters	Unit	Value	
		Icariin	Icariin-HP-γ-cyclodextrin
*T*_max_	H	0.83 ± 0.55	0.75 ± 0.02
*C*_max_	*μ*g/mL	1.84 ± 0.15	9.06 ± 2.02
AUC_0-120_	*μ*g·h/mL	10.69 ± 4.22	213.50 ± 103.12
*t*_1/2_	H	0.81 ± 0.36	6.38 ± 2.07
CL	mL/h/kg	11165 ± 4841.32	645 ± 405

After oral administration, the *C*_max_ and *T*_max_ of icariin in dog plasma were found to be 1.84 *μ*g/mL at 0.83 h for the icariin group and 9.06 *μ*g/mL at 0.75 h for the icariin complex group. AUC_0-120_ was found to be 10.69 *μ*g h/mL in the icariin group and 213.5 *μ*g h/mL in the complex group.

Through complexation with HP-*γ*-cyclodextrin, the water solubility of icariin was increased 654 times, the *C*_*max*_ of icariin was increased about 5 times, the AUC_0-120_ was increased about 20 times, the clearance of icariin was changed from 11165 mL/h/kg to 645 mL/h/kg, the half-life time was changed from 0.81 h to 6.38 h, which indicated that less drug is needed for the same therapeutic effect by using the icariin complex with HP-*γ*-cyclodextrin.

## Conclusion

The inclusion complex of icariin with HP-*γ*-cyclodextrin was prepared by using the solution method through a single factor strategy with addition of a trace amount of water-soluble polymer, and confirmed by FTIR, TAG, DSC, X-ray, SEM and NMR spectral studies. Through the complexation, the water solubility of icariin in the complex was increased by 654 times compared with that of icariin alone, which is the best result for water solubility study of icariin so far, the dissolution rate of icariin was increased by 80 times, and icariin can be 100% released in the first few minutes. *In vivo* pharmacokinetic study showed that the complexation increased *C*_max_ by about 5 times and AUC_0-120_ by about 20 times, shortened the clearance of icariin from 11.17 L/h/kg to 0.65 L/h/kg, extended the half-life of icariin from 0.68 h to 6.38 h, and increased the relative bioavailability by nearly 20 times, indicating that the icariin complex may show better efficacy than icariin alone and become another potential potent hepatoprotective or anti-cancer drug.

## Data Availability

The original contributions presented in the study are included in the article/supplementary material, further inquiries can be directed to the corresponding author.
